# Smoking, development of or recovery from metabolic syndrome, and major adverse cardiovascular events: A nationwide population-based cohort study including 6 million people

**DOI:** 10.1371/journal.pone.0241623

**Published:** 2021-01-12

**Authors:** Sehoon Park, Kyungdo Han, Soojin Lee, Yaerim Kim, Yeonhee Lee, Min Woo Kang, Sanghyun Park, Yong Chul Kim, Seung Seok Han, Hajeong Lee, Jung Pyo Lee, Kwon Wook Joo, Chun Soo Lim, Yon Su Kim, Dong Ki Kim

**Affiliations:** 1 Department of Biomedical Sciences, Seoul National University College of Medicine, Seoul, Korea; 2 Department of Internal Medicine, Armed Forces Capital Hospital, Gyeonggi-do, Korea; 3 Department of Statistics and Actuarial Science, Soongsil University, Seoul, Korea; 4 Department of Internal Medicine, Seoul National University Hospital, Seoul, Korea; 5 Department of Internal Medicine, Seoul National University College of Medicine, Seoul, Korea; 6 Department of Internal Medicine, Keimyung University School of Medicine, Daegu, Korea; 7 Department of Medical Statistics, College of Medicine, Catholic University of Korea, Seoul, Korea; 8 Kidney Research Institute, Seoul National University, Seoul, Korea; 9 Department of Internal Medicine, Seoul National University Boramae Medical Center, Seoul, Korea; Karolinska Institutet, SWEDEN

## Abstract

Smoking, metabolic syndrome (MetS), and major adverse cardiovascular events (MACEs) are important global health problems. We aimed to investigate the association between smoking, alteration in MetS status, and the consequent risk of MACE. We performed a nationwide observational cohort study based on the claims database of Korea. We included people with ≥ 3 national health screenings from 2009 to 2013. Total 6,099,717 people, including 3,576,236 nonsmokers, 862,210 ex-smokers, 949,586 light-to-moderate smokers, and 711,685 heavy smokers, at the first health screening, were investigated. First, we performed a logistic regression analysis using smoking status at the first screening as the exposure variable and MetS development or recovery as the outcome variable. Second, we performed a Poisson regression using smoking status at the third screening as the exposure variable and the outcome was risk of incident MACEs. Among those previously free from MetS (N = 4,889,493), 347,678 people developed MetS, and among those who had previous MetS (N = 1,210,224), 347,627 people recovered from MetS. Smoking was related to a higher risk of MetS development [for heavy smokers: adjusted OR 1.71 (1.69 to 1.73)] and a lower probability of MetS recovery [for heavy smokers: adjusted OR 0.68 (0.67 to 0.69)]. Elevated triglycerides was the MetS component with the most prominent association with smoking. The risk for incident MACEs (78,640 events during a median follow-up of 4.28 years) was the highest for heavy smokers, followed in order by light-to-moderate, ex-smokers and nonsmokers, for every MetS status. Therefore, smoking may promote MetS or even hinder recovery from MetS. Smoking cessation should be emphasized to reduce MACE risk even for those without MetS.

## Introduction

Tobacco smoking is related to various health risks, including cancer, lung disease, fractures, and many others [1, 2]. In particular, smoking causes major adverse cardiovascular events (MACEs), which are a leading cause of death globally [3, 4]. Nevertheless, the smoking rates remain high in certain nations, and approximately a billion people currently smoke globally [5].

Metabolic syndrome (MetS) represents a cluster of conventional metabolic risk factors (e.g., central obesity, glucose intolerance, elevated blood pressure, and dyslipidemia) [6] that is another major contributor to the risk of MACEs in the general population, and its prevalence is rising globally [7, 8]. Previous studies focused on the synergistic harm of smoking and MetS on cardiovascular health [9], and it has been suggested that smoking itself may trigger MetS [10–13]. Therefore, controlling metabolic risk factors by reducing smoking and encouraging a healthy diet and exercise is a common lifestyle modification strategy to reduce the risk of MACEs [14–18]. Few large cohort studies that have investigated the association between MetS and MACEs have considered smoking history [9]. Nevertheless, as a patient’s MetS status can change dynamically [19], population-scale evidence obtained in quantitative assessments of the relationship between the risk of MACEs and smoking in people with various dynamic MetS states would be useful information for guiding public policy. In addition, whether smoking is associated with hindrance of recovery from MetS or facilitating its development could be further investigated longitudinally at a large-scale in a nationwide cohort study in which MetS status is assessed sequentially [20]. In particular, determining which MetS component is more prominently related to smoking or whether MetS recovery is inhibited by smoking would be essential to further evaluating the adverse effects of smoking on metabolic health.

In this nationwide study, we assessed one of the largest nationwide cohorts to investigate whether prior smoking status was related to a higher risk of MetS development or to hindrance during recovery from previous MetS. We further analyzed the MACE prognosis of people with various dynamic MetS statuses according to their smoking status. We hypothesized that smoking would be associated with the facilitation of MetS and a higher MACE risk in the general population.

## Materials and methods

### Ethics considerations

The institutional review board of Seoul National University Hospital (no. E-1801-027-913) approved this study. The institutional review board waived the requirement for informed consent as anonymous data from the National Health Insurance Service of Republic of Korea. The data was provided by the National Health Insurance Service after approval (no. NHIS-2019-1-651). The study was conducted in accordance with the Declaration of Helsinki.

### Study setting

The study environment was described in our previous study [19]. The National Health Insurance Service of the Republic of Korea provides general nationwide health insurance services for all Korean people, and the related claims information and health screening results are accumulated in a public database [21, 22]. General health screening includes a free-of-charge nationwide exam provided to subscribers. We used the self-reported questionnaire results included in the health screening to evaluate smoking exposure, the lab data obtained in health screenings and claims information to evaluate MetS status, and claims information to identify MACE outcomes.

### Study population

**Fig 1:**
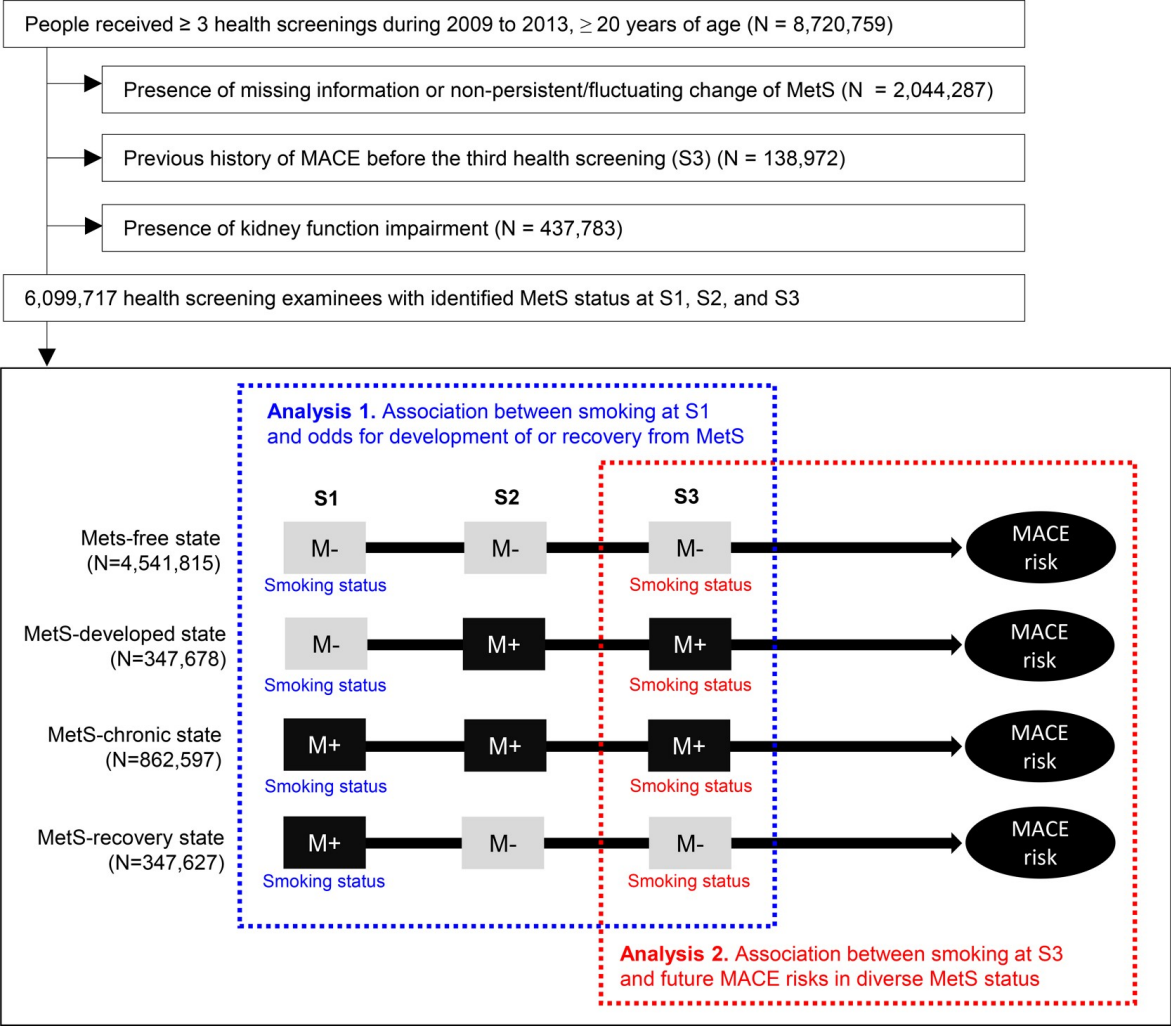
Study population. The flow diagram for the study population. The first three health screenings are indicated as S1, S2, and S3. The gray squares with “M‒” indicate a health screening without identified metabolic syndrome MetS. Black squares with “M+” indicate a health screening with identified MetS. The first part of the study was performed with the smoking status at S1 used as the exposure variable and change in MetS status between S2 and S3 compared to S1 as the outcome variable. The second part of the study was performed with smoking status at S3 used as the exposure variable and future risk of MACE used as the outcome variable. MetS = metabolic syndrome, MACE = major adverse cardiovascular event.

We included adults (≥ 20 years old) who received ≥ 3 national health screenings from 2009 to 2013 (Fig 1), and the information from the first three health screenings (S1, S2, and S3) and the associated claims data were investigated to obtain the study population. As we could not include persons with insufficient information related to lifestyle variables, clinical characteristics, or MetS profiles, those with missing information in the analyzed variables were excluded. In addition, we excluded persons with transient changes in MetS status as we aimed to study subjects who showed stable changes in or the maintenance of their MetS state. In addition, we also excluded those showing changes in MetS status at the third health screening (S3) to ensure that the identified changes in MetS status were consistent. Finally, as we intended to study a population with no history of MACEs or significant kidney function impairment before the completion of the first 3 health screenings (S1, S2, and S3), we excluded persons with a history of MACE or indicators of kidney diseases (baseline estimated glomerular filtration rate < 60 mL/min/1.73 m^2^ calculated using the Modification of Diet in Renal Disease equation, a chronic kidney disease diagnostic code, or a history of renal replacement therapy) before S3. Exclusion of those with kidney function impairment was because the clinical significance of metabolic parameters may be different in the people with chronic kidney disease [23, 24].

### Data collection

The main exposure variable was smoking status, which was collected from self-reported questionnaires included in the general health screening, which can be found in S1 Fig in S1 File. Non-smokers were those who had not smoked a total of ≥ 100 cigarettes in their lifetime and were not smoking at the time, ex-smokers as those who smoked ≥ 100 cigarettes in their lifetime but were not smoking at the time, and current-smokers as those who were smoking at the time of the survey [25]. We additionally stratified the current-smokers as “light-to-moderate” and “heavy” smokers, and those who smoked ≥ 20 cigarettes per day were defined as heavy smokers). Other details related to demographics, lifestyle variables, and variables that reflect the severity of MetS are presented in S2 Method in S1 File. All characteristics, including smoking status, were collected at both S1 and S3.

### Identification of MetS status

MetS status was determined using the harmonized criteria, as previously described [6]. People with ≥ 3 of the 5 following criteria were considered to have MetS at the corresponding health screening: central obesity (waist circumference ≥ 90 cm for males and ≥ 80 cm for females among Asian individuals); high triglycerides (≥ 150 mg/dL or the use of a relevant drug); low high-density lipoprotein cholesterol (< 40 mg/dL for males, 50 mg/dL for females or the use of a relevant drug); high blood pressure (systolic ≥ 130 and/or diastolic ≥ 80 mmHg or the use of an anti-hypertensives); and high fasting plasma glucose (fasting glucose ≥ 100 mg/dL or the use of an anti-diabetics). A free state of a metabolic component was defined when a relevant medication was not prescribed and the lab or anthropometric value was not in the abnormal ranges.

Based on the absence/presence state of Mets in the first three health screenings (S1, S2, and S3), we defined four dynamic MetS statuses (Fig 1): MetS-free, when no MetS (< 3 MetS components) was found during S1, S2, and S3; MetS-chronic, when MetS was present for all of S1, S2, and S3; MetS-recovery, when stable recovery from preexisting MetS was observed (MetS present at S1 but not at S2 and S3); and MetS-developed, when MetS was newly developed and persistent for one more health screening (no MetS at S1 but MetS present at S2 and S3) [19].

These 4 MetS statuses were used as the outcome variable in the analysis of the association between smoking status at S1 and the probability of the MetS status changing during the S2 and S3 follow-up screenings. In addition, MetS status was used to define subgroups in the analysis of the associations between smoking status at S3 and future MACE risks in people with various MetS statuses.

### Assessment of the association between smoking and metabolic syndrome

In the first part of this study, we used smoking status at S1 as the exposure variable and change in MetS status as the outcome variable (Fig 1). In this investigation, the first outcome was “development of MetS”, which was studied in patients free from MetS at S1 (the “MetS-developed” and “MetS-free” status groups described above). Those with a MetS-developed state were considered to have the outcome occurrence. The second outcome was “recovery from MetS”, which included people with MetS at S1 (those with a “MetS-recovered” or “MetS-chronic” status). People with a MetS-recovery state included those with the recovery outcome.

We additionally performed analyses regarding the association between smoking status and the probability of each MetS component (central obesity, elevated blood pressure, high fasting plasma glucose, high triglycerides, and low high-density lipoprotein cholesterol). In these analyses, we defined the dynamic status of each MetS component following the same method described above, and the odds for development of or recovery from each MetS component were investigated according to smoking status at S1.

### Assessment of the association between smoking and MACEs in various MetS statuses

In the second part of the study, smoking status at S3 was used as the exposure variable, and future risk of MACEs after completion of the three health screenings (S1, S2, and S3) was the outcome variable. In this analysis, we investigated these associations by dividing each subgroup according to dynamic MetS status (Fig 1). The prognostic study outcome, MACE, was a composite of acute myocardial infarction, coronary revascularization and acute ischemic stroke events, which were defined according the claims database, as described in S2 Method in S1 File. The follow-up was initiated from a day after S3. We censored death events or follow-up ended on December 31, 2016, as no additional claims information was available for this study after that date.

### Statistical analysis

Continuous variables are presented as mean values (standard deviations), and categorical variables are presented as numbers (percentages). For the associations between prior smoking status and MetS development or recovery, odds ratios for the outcomes were calculated with logistic regression analyses. For the associations between smoking status and MACE risk in the people with various MetS statuses, the incidence rate ratios of MACEs were calculated with Poisson regression. The age- and sex-adjusted results are presented as smoking exposure was substantially different according to these two important clinical variables. The details regarding the constructions and statistical analysis of the multivariable regression model are described in S2 Method in S1 File. All statistical analyses were performed using SAS, and two-sided P values <0.05 were considered significant.

## Results

### Study population and their characteristics

Among the 8,720,759 people who had ≥ 3 health screenings, we excluded those with missing information or nonstable MetS status (N = 2,044,287), those with a history of MACEs before the third health screening (S3) (N = 138,972), and those with indicators of kidney function impairment (N = 437,782). We finally included 6,099,717 people in this study (Fig 1).

The characteristics of the study population were collected at S1 and S3. At the first health screening (S1), 3,576,236 non-smokers, 862,210 ex-smokers, 949,586 current light-to-moderate smokers, and 711,685 current heavy smokers were identified (Table 1). The current smokers were predominantly male and relatively younger than ex-smokers or nonsmokers. Among nonsmokers, a relatively large portion of them was female, and the proportion not engaged in moderate-to-vigorous physical activity or alcohol consumption was higher than the proportion that was engaged in these activities. Regarding the MetS components present at S1, the proportions of nonsmokers and light-to-moderate current smokers who were free from any MetS component were relatively higher than the corresponding proportion of heavy or ex-smokers. The overall trend was similar when the characteristics were collected at the third health screening (S3) (S3 Table in S1 File).

**Table 1:** Characteristics of the study population at the first health screening (S1).

Variables	Nonsmoker	Ex-smoker	Current, light-to-moderate	Current, heavy
**Number of people**	3576236	862210	949586	711685
**Clinical and demographic characteristics**				
Age (years)	44.9±13.5	46.3±12.2	38.3±11.6	42.5±11.0
Sex (male)	1107721 (31)	825458 (96)	885714 (93)	703703 (99)
Height (cm)	161.2±8.5	169.8±6.5	170.9±6.9	170.6±6.3
Weight (kg)	60.1±10.5	69.5±9.9	68.5±11.0	69.7±11.0
BMI (kg/m^2^)	23.1±3.2	24.1±2.9	23.4±3.1	23.9±3.2
Low-income status	892599 (25)	129771 (15)	158267 (17)	121887 (17)
CCI (score)	0.7±1.1	0.7±1.1	0.4±0.8	0.5±1.0
Hemoglobin (g/dL)	13.4±1.5	14.8±1.2	15.0±1.2	15.1±1.2
AST (IU/L)	21.8±24.2	28.7±28.5	27.7±32.8	30.0±30.7
ALT (IU/L)	23.5±20.0	27.0±24.2	25.8±26.2	27.3±26.3
Cr (mg/dL)	0.99±1.08	1.21±1.38	1.21±1.4	1.16±1.25
eGFR (mL/min/1.73 m^2^)	89.6±40.8	88.1±50.5	91.4±50.1	90.8±44.2
**Self-reported lifestyle at S1**				
Moderate-to-vigorous activity				
None	1828394 (51)	283577 (33)	372379 (39)	336808 (47)
1–2 days/wk	958532 (27)	309023 (36)	366936 (39)	234731 (33)
3–4 days/wk	480100 (13)	169986 (20)	137231 (14)	88056 (12)
≥ 5 days/wk	309210 (9)	99624 (12)	73040 (8)	52090 (7)
Alcohol				
No alcohol intake	2313657 (65)	229686 (27)	199396 (21)	155518 (22)
Moderate consumption	175131 (5)	57088 (7)	47067 (5)	27933 (4)
Heavy consumption	1087448 (30)	575436 (67)	703123 (74)	528234 (74)
**Parameters of MetS at S1**				
Waist circumference (cm)	77.0±9.0	83.1±7.7	80.8±8.1	82.8±8.1
Systolic BP (mmHg)	119.3±14.5	123.8±13.6	121.5±13.0	123.2±13.5
Diastolic BP (mmHg)	74.4±9.7	77.7±9.5	76.3±9.2	77.5±9.5
Glucose (mg/dL)	94.0±19.0	98.1±22.1	94.0±19.9	97.9±25.4
Triglycerides (mg/dL)	110.7±78.7	142.1±104.3	141.5±105.4	165.0±124.3
HDL cholesterol (mg/dL)	58.3±21.0	54.3±19.4	54.3±17.9	52.6±19.3
**No. of MetS components at S1**				
0	1264746 (35)	213375 (25)	313231 (33)	171913 (24)
1	1022679 (29)	255088 (30)	300290 (32)	208881 (29)
2	605222 (17)	189524 (22)	184948 (19)	159545 (22)
3	358484 (10)	116308 (13)	95750 (10)	101153 (14)
4	226987 (6)	66504 (8)	43842 (5)	53983 (8)
5	98118 (3)	21411 (2)	11525 (1)	16210 (2)

MetS = metabolic syndrome, BMI = body mass index, CCI = Charlson Comorbidity Index, AST = aspartate aminotransferase, ALT = alanine aminotransferase, Cr = creatinine, eGFR = estimated glomerular filtration rate, BP = blood pressure, HDL = high-density lipoprotein.

There were no missing values in the table.

### Prior smoking status and development of or recovery from MetS

The numbers of people with dynamic or stable MetS status were as follows: MetS-free [N = 4,541,815 (74.3%)], MetS-chronic [N = 862,597 (13.5%)], MetS-developed [N = 347,627 (5.7%)], and MetS-recovery [N = 347,678 (5.7%)]. Although the odds ratios were heterogeneous in the unadjusted model, people with a smoking history at the first screening had a significantly higher risk of MetS development and a lower probability of recovering from MetS in the age- and sex-adjusted models (Table 2). In the multivariable model adjusted for variables including body mass index, severity of MetS, physical activity and alcohol consumption behavior, being an ex-smoker was related to approximately 10% higher odds of developing MetS and 10% lower odds of MetS recovery than was found in nonsmokers. Current smokers at S1 had higher probabilities of having MetS at S2 and S3 than was found in ex-smokers. In particular, heavy smoking was associated with a more than 70% higher odds of developing MetS and a more than 30% lower odds of recovering from MetS than was found in nonsmokers.

**Table 2:** Smoking status and its association with recovery from or development of metabolic syndrome.

Prior MetS status and outcome	Smoking status at S1	Number of subjects	Number of outcomes and percent	Unadjusted model		Model 1. Age-/sex-adjusted		^a^Model 2. Clinical factors, including the values of adjusted MetS parameters		^b^Model 3. Clinical factors, including the number of MetS components and other adjusted lifestyle variables	
				OR (95% CI)	*p*	Adjusted OR (95% CI)	*p*	Adjusted OR (95% CI)	*p*	Adjusted OR (95% CI)	*p*
MetS development as the outcome in persons without underlying MetS (MetS-free or MetS-developed state)	Nonsmoker	2892647	191211 (6.6%)	Reference		Reference		Reference		Reference	
	Ex-smoker	657987	54702 (8.3%)	1.28 (1.27–1.29)	< 0.001	1.20 (1.18–1.21)	< 0.001	1.08 (1.07–1.10)	< 0.001	1.10 (1.08–1.11)	< 0.001
	Current, light-to-moderate	798469	48887 (6.1%)	0.92 (0.91–0.93)	< 0.001	1.32 (1.30–1.34)	< 0.001	1.33 (1.31–1.34)	< 0.001	1.37 (1.35–1.38)	< 0.001
	Current, heavy	540339	52827 (9.8%)	1.53 (1.52–1.55)	< 0.001	1.80 (1.78–1.82)	< 0.001	1.61 (1.59–1.63)	< 0.001	1.71 (1.69–1.73)	< 0.001
MetS-recovery as the outcome in persons with underlying MetS (MetS-chronic or MetS-recovery state)	Nonsmoker	683589	181169 (26.5%)	Reference		Reference		Reference		Reference	
	Ex-smoker	204223	61372 (30.0%)	1.19 (1.18–1.20)	< 0.001	0.88 (0.87–0.89)	< 0.001	0.89 (0.88–0.90)	< 0.001	0.90 (0.89–0.92)	< 0.001
	Current, light-to-moderate	151117	54630 (36.2%)	1.57 (1.55–1.59)	< 0.001	0.86 (0.85–0.88)	< 0.001	0.84 (0.83–0.86)	0.001	0.82 (0.81–0.84)	< 0.001
	Current, heavy	171346	50507 (29.5%)	1.16 (1.15–1.17)	< 0.001	0.68 (0.67–0.69)	< 0.001	0.72 (0.71–0.74)	< 0.001	0.68 (0.67–0.69)	< 0.001

MetS = metabolic syndrome, OR = odds ratio, CI = confidence interval.

Heavy smoking was defined as smoking ≥ 20 cigarettes per day.

^a^Model 2 was adjusted for age, sex, baseline eGFR (continuous, mL/min/1.73 m^2^), alanine aminotransferase (continuous, IU/mL aspartate aminotransferase (continuous, IU/mL), hemoglobin (continuous, g/dL), low income status (the lowest quartile in the nation), body mass index (continuous, kg/m^2^), the Charlson Comorbidity Index, and the values of MetS components at the first health screening (S1).

^b^Model 3 was adjusted for the same variables as Model 2, but the number of MetS components at the first screening (S1) was adjusted for instead the values of MetS components and other lifestyle variables [self-reported frequencies of moderate-to-vigorous exercise (categorical: none/week, 1–2 days/week, 3–4 days/week, and ≥ 5 days/week) and drinking status (categorical: none, moderate consumption of ≤ 2 standard drinks for males and ≤ 1 standard drink for females per single drinking session, and more than moderate)] were additionally adjusted for the model.

**Fig 2:**
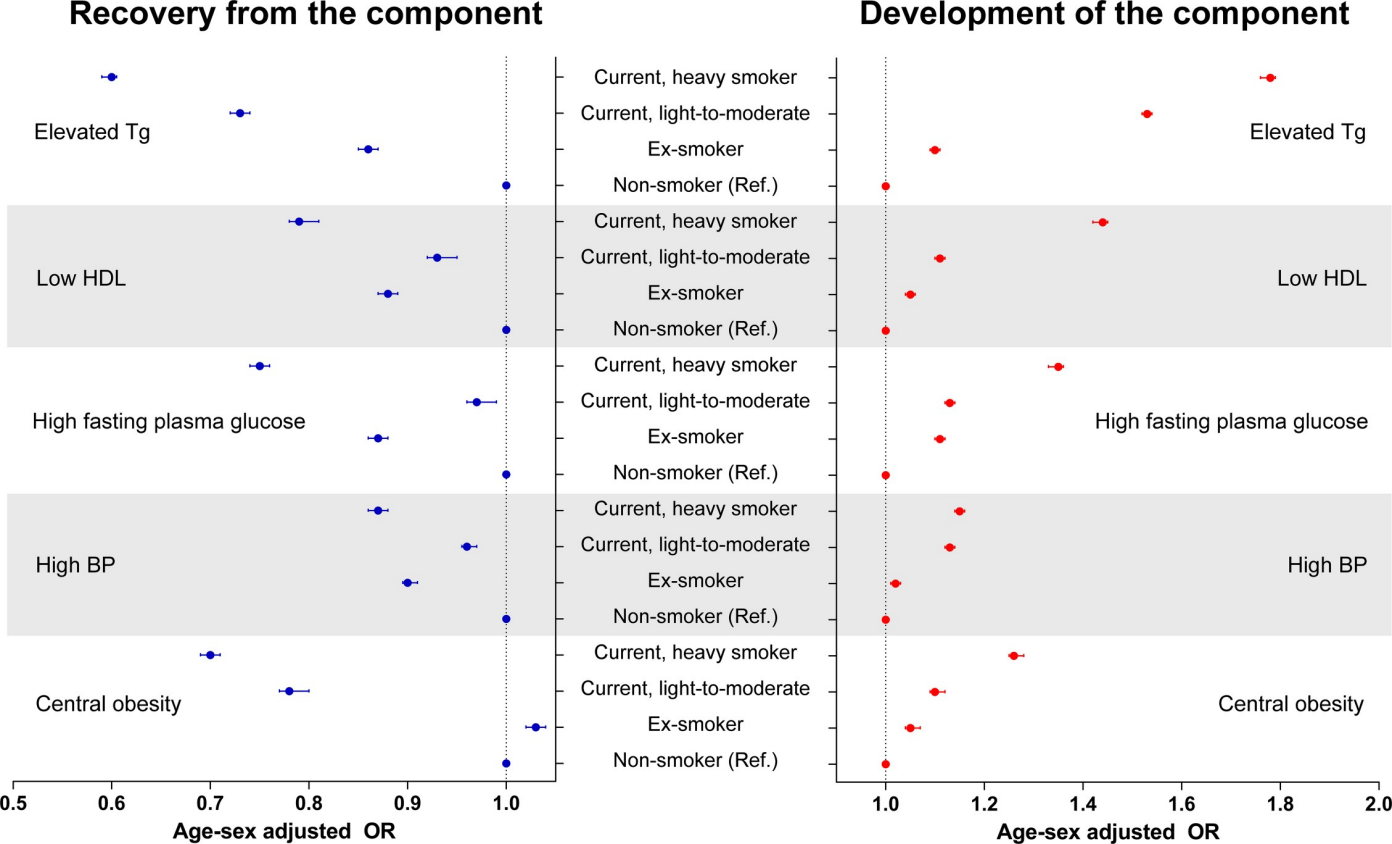
Association between smoking status and development of or recovery from individual MetS components. The blue dots and error bars indicate the age- and sex-adjusted odds ratios and 95% confidence intervals for recovery from the component. The red dots and error bars indicate the age- and sex-adjusted odds ratios and 95% confidence intervals for the development of the component. The recovery from individual MetS components was assessed within between individuals with the component at the first health screening (S1) and those who no longer fulfilled the criterion in the second (S2) and third (S3) health screenings were considered to be recovered from the MetS component. The development of individual MetS components was assessed between individuals without the component at S1 and those who fulfilled the criterion at S2 and S3 were considered to have developed the MetS component. All odds ratios are presented with nonsmokers used as the reference group.

Regarding individual components, we found that elevated triglycerides was most strongly associated with smoking status after considering the association sizes with regard for both the development of and recovery from the component (Fig 2). This finding remained similar when other characteristics, including self-reported frequencies of moderate-to-vigorous physical activity or alcohol consumption, were also adjusted (S4 and S5 Tables in S1 File). The development of or recovery from other MetS components were also significantly associated with prior smoking status, and current smoking was associated with higher odds of future MetS, particularly in heavy smokers, in the multivariable analysis.

### MACE risks according to smoking status in MetS subgroups

**Fig 3:**
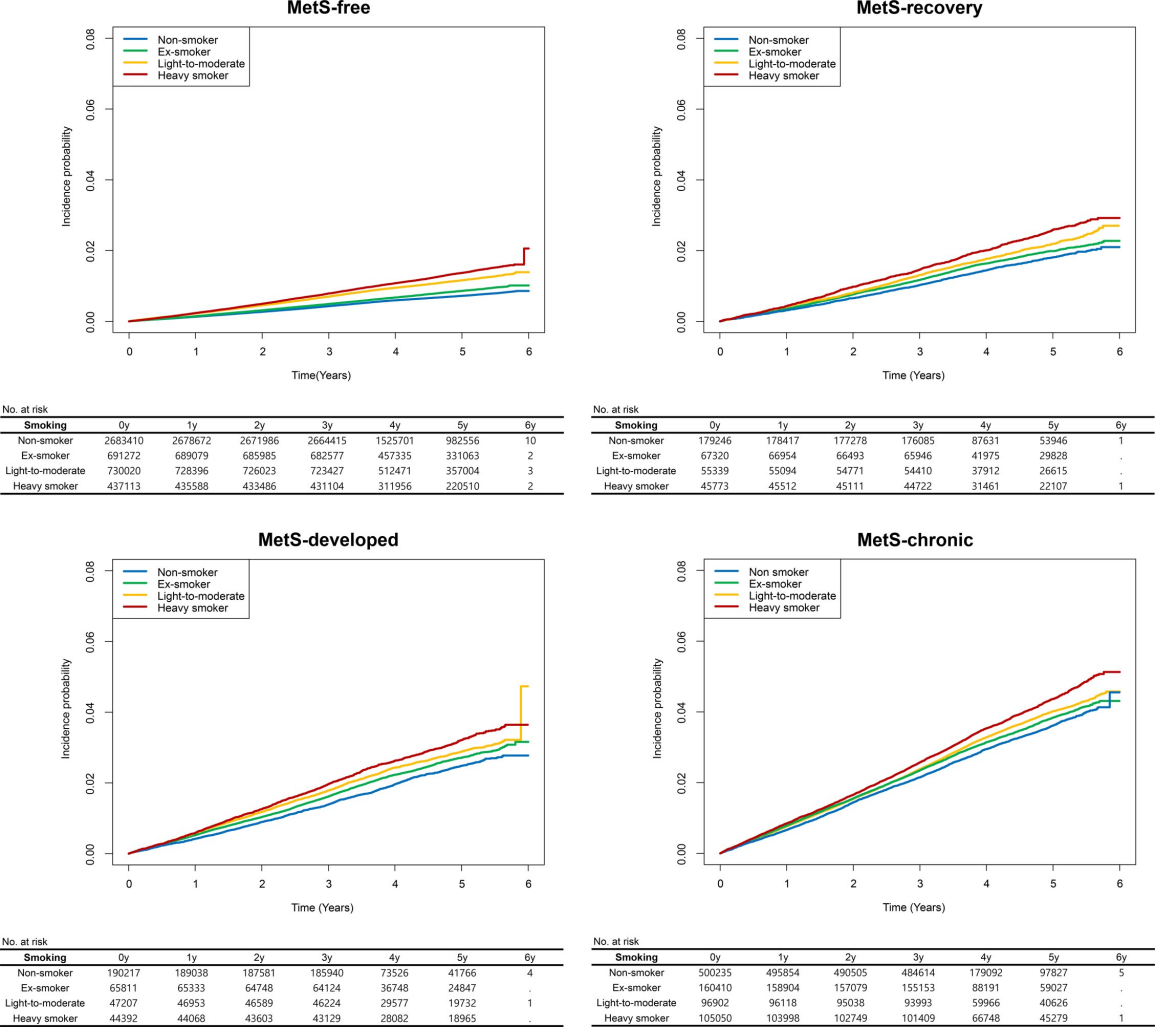
The survival curves were plotted in each MetS subgroup. The y-axes indicate the incidence probability of MACEs, including acute myocardial infarction, revascularization, and acute ischemic stroke. The x-axes indicate the time (years). The blue, green, orange and red curves indicate the incidences of MACEs in nonsmokers, ex-smokers, light-to-moderate smokers, and heavy smokers, respectively. The tables below the survival curves show the numbers of persons at risk. MetS = metabolic syndrome.

The study population was followed-up for a median of 4.28 (3.50–5.25) years after the three health screenings. The incidence rate ratios for MACEs in each MetS subgroup are shown according to smoking status at S3 in Table 3 and differed significantly among the groups, plotted in Fig 3. Heavy smokers in the MetS-chronic state had the highest (8.94/1,000 person-years), while nonsmokers in the MetS-free state had the lowest (1.46/1,000 person-years) incidence rate of MACEs. The age- and sex-adjusted risks of MACEs were found, in increasing order, in non-smokers, ex-smokers, light-to-moderate current smokers, and heavy smokers, with heavy smokers having a nearly 2-fold higher risk than was found in nonsmokers. Although those with MetS at S3 had a higher risk of a MACE than was found in those without, those who were smoking but in a MetS-free or MetS-recovery state had a comparable or even higher adjusted risk of MACEs than was found in those who were not smoking and in a MetS-developed or MetS-chronic state. Similar results were found for acute myocardial infarction, revascularization, and acute ischemic stroke outcomes when analyzed individually (S6, S7, and S8 Tables in S1 File). When additional adjustments for other clinicodemographic characteristics and baseline severity of MetS were performed, an ex-smoker history was related to an approximately 5–10% higher risk of MACEs in each MetS status subgroup (Table 4). In addition, the light-to-moderate current smokers and heavy smokers had an approximately 50% and 90% higher risk of MACEs, respectively, than was found in nonsmokers, regardless of MetS status.

**Table 3:** Risks of major adverse cardiovascular events according to smoking status at S3 and dynamic metabolic syndrome status.

MetS status	Smoking status at S3	Number of subjects	Number of MACEs	Follow-up (person-years)	Incidence rate (/1000 person-years)	Unadjusted model		Age-/sex-adjusted	
						IRR (95% CI)	*p*	Adjusted IRR (95% CI)	*p*
MetS-free	Nonsmoker	2683410	16892	11587438	1.46	1 (Ref.)	< 0.001	1 (Ref.)	< 0.001
	Ex-smoker	691272	7328	3119304	2.35	1.61 (1.57–1.66)	< 0.001	1.08 (1.05–1.11)	< 0.001
	Current, light-to-moderate	730020	5705	3324731	1.72	1.18 (1.14–1.21)	< 0.001	1.43 (1.38–1.47)	< 0.001
	Current, heavy	437113	5474	1994115	2.75	1.88 (1.83–1.94)	< 0.001	1.86 (1.80–1.92)	< 0.001
MetS-recovery	Nonsmoker	179246	3020	749040	4.03	2.77 (2.66–2.87)	< 0.001	1.47 (1.41–1.53)	< 0.001
	Ex-smoker	67320	1337	298000	4.49	3.08 (2.91–3.25)	< 0.001	1.57 (1.48–1.66)	< 0.001
	Current, light-to-moderate	55339	905	249434	3.63	2.49 (2.33–2.66)	< 0.001	2.11 (1.98–2.26)	< 0.001
	Current, heavy	45773	1060	205848	5.15	3.53 (3.32–3.76)	< 0.001	2.86 (2.68–3.04)	< 0.001
MetS-developed	Nonsmoker	190217	4213	761886	5.53	3.79 (3.67–3.92)	< 0.001	1.67 (1.62–1.73)	< 0.001
	Ex-smoker	65811	1671	281987	5.93	4.06 (3.87–4.27)	< 0.001	1.80 (1.71–1.90)	< 0.001
	Current, light-to-moderate	47207	1019	206241	4.94	3.39 (3.18–3.61)	< 0.001	2.61 (2.45–2.79)	< 0.001
	Current, heavy	44392	1273	193887	6.57	4.50 (4.25–4.77)	< 0.001	3.43 (3.24–3.64)	< 0.001
MetS-chronic	Nonsmoker	500235	16170	1977354	8.18	5.61 (5.49–5.73)	< 0.001	2.03 (1.98–2.07)	< 0.001
	Ex-smoker	160410	5362	683130	7.85	5.38 (5.22–5.55)	< 0.001	2.08 (2.01–2.15)	< 0.001
	Current, light-to-moderate	96902	3110	421703	7.37	5.06 (4.87–5.26)	< 0.001	3.18 (3.05–3.30)	< 0.001
	Current, heavy	105050	4101	458713	8.94	6.13 (5.93–6.35)	< 0.001	4.05 (3.91–4.20)	< 0.001

MetS = metabolic syndrome, MACE = major adverse cardiovascular events, IRR = incidence rate ratio, CI = confidence interval

Heavy smoking was defined as smoking ≥ 20 cigarettes per day.

**Table 4:** Risk of major adverse cardiovascular events according to smoking status at the third health screening (S3) in each study group.

MetS status, and smoking exposure at S3	^a^Model 1. Baseline severity of MetS (number of MetS components)-adjusted		^b^Model 2. Baseline severity of MetS (laboratory parameters of MetS)-adjusted		^c^Model 3. Baseline severity of MetS (number of MetS components)- and other lifestyle variables (smoking, alcohol)-adjusted	
	Adjusted IRR (95% CI)	P	Adjusted IRR (95% CI)	P	Adjusted IRR (95% CI)	P
MetS-free						
Nonsmoker	1 (Ref.)	< 0.001	1 (Ref.)	< 0.001	1 (Ref.)	< 0.001
Ex-smoker	1.04 (1.01–1.08)	0.007	1.07 (1.04–1.10)	< 0.001	1.07 (1.04–1.11)	< 0.001
Current, light-to-moderate	1.48 (1.43–1.53)	< 0.001	1.53 (1.48–1.59)	< 0.001	1.51 (1.46–1.56)	< 0.001
Current, heavy	1.84 (1.78–1.91)	< 0.001	1.96 (1.89–2.03)	< 0.001	1.86 (1.80–1.93)	< 0.001
MetS-recovery						
Nonsmoker	1 (Ref.)	< 0.001	1 (Ref.)	< 0.001	1 (Ref.)	< 0.001
Ex-smoker	1.05 (0.97–1.13)	0.196	1.07 (1.00–1.16)	0.066	1.11 (1.02–1.19)	0.010
Current, light-to-moderate	1.44 (1.33–1.57)	< 0.001	1.47 (1.35–1.60)	< 0.001	1.52 (1.39–1.65)	< 0.001
Current, heavy	1.85 (1.71–2.01)	< 0.001	1.93 (1.77–2.09)	< 0.001	1.93 (1.77–2.10)	< 0.001
MetS-developed						
Nonsmoker	1 (Ref.)	< 0.001	1 (Ref.)	< 0.001	1 (Ref.)	< 0.001
Ex-smoker	1.02 (0.95–1.09)	0.570	1.04 (0.97–1.11)	0.269	1.07 (1.00–1.14)	0.064
Current, light-to-moderate	1.44 (1.33–1.56)	< 0.001	1.45 (1.34–1.57)	< 0.001	1.51 (1.39–1.63)	< 0.001
Current, heavy	1.82 (1.69–1.96)	< 0.001	1.84 (1.70–1.98)	< 0.001	1.88 (1.74–2.03)	< 0.001
MetS-chronic						
Nonsmoker	1 (Ref.)	< 0.001	1 (Ref.)	< 0.001	1 (Ref.)	< 0.001
Ex-smoker	1.00 (0.96–1.04)	0.943	1.02 (0.98–1.06)	0.335	1.05 (1.01–1.10)	0.008
Current, light-to-moderate	1.45 (1.38–1.51)	< 0.001	1.44 (1.38–1.51)	< 0.001	1.52 (1.45–1.59)	< 0.001
Current, heavy	1.74 (1.66–1.81)	< 0.001	1.72 (1.65–1.79)	< 0.001	1.80 (1.73–1.88)	< 0.001

MetS = metabolic syndrome, IRR = incidence rate ratio, CI = confidence interval.

^a^Model 1 was adjusted for age, sex, baseline eGFR (continuous, mL/min/1.73 m^2^), alanine aminotransferase levels (continuous, IU/mL), aspartate aminotransferase levels (continuous, IU/mL), hemoglobin levels (continuous, g/dL), low income status (the lowest quartile in the nation), body mass index, the Charlson Comorbidity Index, and the number of MetS components at the third health screening (S3).

^b^Model 2 was adjusted for the same variables as those used in Model 1, but the exact values obtained for waist circumference, systolic BP, diastolic BP, fasting glucose levels, triglyceride levels, and high-density lipoprotein cholesterol levels were included in the multivariable model instead of the number of MetS components.

^c^Model 3 was adjusted for the same variables as those used in Model 1, but the other lifestyle variables (smoking: none, previous, or current; and alcohol consumption: none, moderate, or heavy) at S3 were additionally included in the adjustment variable.

## Discussion

In this nationwide study, smoking was significantly associated with the development or hindrance of recovery from MetS. Elevated triglycerides was the MetS criterion for which the development or hindrance of recovery was most prominently associated with smoking status. In addition, regardless of which MetS status group was analyzed, a smoking history was related to a higher MACE risk, and current smokers, especially heavy smokers, had an even higher risk of MACE. Our study provides large-scale evidence showing that smoking may cause harm during all stages of cardiovascular disease development, from the facilitation of MetS to a higher risk of MACEs.

A population-scale study investigating whether smoking promotes MetS including sequential health screening results is scarce. Moreover, a description of the sizes of the associations between smoking and MACE risks in the general population with diverse MetS statuses would be helpful as MACE risks vary substantially according to the population’s dynamic MetS status [19]. Our study has strengths, including that we found 1) that smoking may promote or hinder recovery from MetS in a large-scale general population cohort including repetitive assessments for MetS; 2) that the sizes of association with smoking varied among the MetS components, with elevated triglycerides having the strongest association; 3) and that the association between smoking and MACE risks was significant regardless of MetS status. Our study results additionally alert the medical community to the harm caused by smoking at overall stages from MetS to MACE development and further emphasize the importance of smoking cessation.

Few studies have explored cross-sectional associations between smoking and MetS prevalence [11–13], and some studies have shown that smoking is related to higher risks of incident MetS components, providing important insights into the adverse effects of smoking on metabolic health [20, 26–31]. The previous studies firstly focused on the impact of smoking on the development of diabetes, and a previous meta-analysis investigated 1.2 million individuals and reported that the risk of diabetes associated with active smoking [20]. In Korea, a prospective cohort study including 1.2 million individuals also reported a significant association between smoking and diabetes [28]. There were other studies demonstrating that smoking is associated with the risk of hypertension [29, 30], obesity [31], and dyslipidemia [12]. Our study results additionally confirm previous reports that showed that smoking may worsen metabolic profiles; in this study, our large-scale repetitive assessment of various MetS profiles including 6 million people emphasizes the finding. In addition, our finding that smoking may suppress MetS recovery, which commonly occurs in persons with relatively nonsevere MetS [19], is important because few population-based cohort studies have explored this issue. In our study results, current smokers had higher adjusted probabilities of MetS than was found in ex- or nonsmokers, and heavy smokers had the highest risks of MetS development and the lowest probability of recovery from MetS. Therefore, people with metabolic risk factors and smoking histories should be made aware of these risks and encouraged to try to quit or at least reduce smoking to ameliorate the critical burden of MetS.

Our study shows that all individual MetS components were associated with smoking, additionally emphasizing the harm caused by smoking on metabolic health. Additionally, we compared the sizes of the associations between smoking and individual MetS components and found that elevated triglycerides or low high-density lipoprotein cholesterol, the dyslipidemia, had relatively large association sizes [12, 32]. These results suggest that further studies focused on the mechanisms of dyslipidemia in smokers are warranted.

The age- and sex-adjusted effect sizes of the associations between smoking and MACE risks are noteworthy. Heavy smokers consistently free of MetS had even higher risks of MACE than were found in those who developed MetS but were not smoking, further supporting the notion that smoking alone exerts adverse effects. Also, the finding suggests the possibility that quitting smoking may overcome the adverse impact of MetS in certain people, emphasizing the necessity of smoke cessation. As the age- and sex-adjusted incidence rate of MACE was approximately 90% higher in heavy smokers than in nonsmokers, this dangerous behavior should be particularly discouraged. In addition, even people with a MetS-free or MetS-recovery state had significantly higher risks of MACEs if they were smokers, so these metabolically healthy smokers should not be considered assured due to their absence of MetS and smoking should be similarly discouraged regardless of absence of MetS [33].

The current study which reported both the association between smoking and MetS, and smoking and MACE in various MetS status provides an integrative view for the adverse association between smoking, MetS, and MACE. Our findings suggest that smoking may have a harmful effect on the longitudinal spectrum of MACE development, from the initial development of MetS. In addition, the results demonstrated that smoking and MetS might have synergistic harm on MACE. Although the causality should be proven in further studies, policy to reduce the smoking rate would be one of the fundamental approaches to decrease the crucial burden of MetS and MACE in the current medicine.

There are several limitations in this study. First, as this is a retrospective study, there may be hidden confounders or unincluded events. In particular, consideration of nutrition may have provided additional insights; however, as this variable is not collected in general health screenings. Second, the accuracy of the implemented questionnaire has not been validated. However, as the questions included in the survey are relatively simple and the method used to quantify smoking history followed general consensus, limitations related to the questionnaire form may be not critical. Third, the study population included individuals residing in a single nation, and their ethnic backgrounds were mostly Asian. Although this is a rare large-scale Asian cohort that assessed important health variables, whether the study results can be applied to populations in other nations is not secured. Finally, the smokers were predominantly male, similar to the actual smoking proportions reported from 2009 to 2013 in Korea, where more than 40% of male subjects and fewer than 10% of female subjects are smokers. As the main age- and sex-adjusted results and further multivariable models showed consistent trends, we believe this limitation is not critical, but it may be necessary to perform additional investigations in nations with higher female smoking rates.

In conclusion, smoking may facilitate MetS development or inhibit recovery from MetS, further emphasizing that reducing the smoking rate is essential to alleviating the critical burden of MetS in general population. In addition, smoking was associated with higher MACE risks in general people with different MetS statuses, even those free from MetS. Cessation of smoking should be emphasized in general population to decrease the future burden of MetS and the risks of MACEs.

## Supporting information

S1 File (DOCX)
