# Supplementary material for: Smoking, development of or recovery from metabolic syndrome, and major adverse cardiovascular events: A nationwide population-based cohort study including 6 million people
Source: PLoS One. 2021 Jan 12;16(1):e0241623. doi: 10.1371/journal.pone.0241623 (PMC7802921; doi:10.1371/journal.pone.0241623)
Supplement: S1 File — (DOCX) [file pone.0241623.s001.docx]

**Supporting Information**

**S1 Fig. English version questionnaire used in general health screenings by the National Health Insurance Service of Korea.**

**S2 Method. Additional information regarding the methods.**

**S3 Table. Characteristics of the study population at the third health screening (S3).**

**S4 Table. Association of smoking status and the development of a component of MetS in individuals who were free from the component at the first health screening (S1).**

**S5 Table Association between smoking status and recovery from a component of MetS in individuals previously with the component at the first health screening (S1).**

**S6 Table. Risk of acute myocardial infarction according to smoking at the third health screening (S3) and dynamic metabolic syndrome status.**

**S7 Table. Risk of coronary revascularization according to smoking at the third health screening (S3) and dynamic metabolic syndrome status.**

**S8 Table. Risk of acute ischemic stroke according to smoking at the third health screening (S3) and dynamic metabolic syndrome status.**

**S1 Fig. English version questionnaire used in general health screenings by the National Health Insurance Service of Korea.**


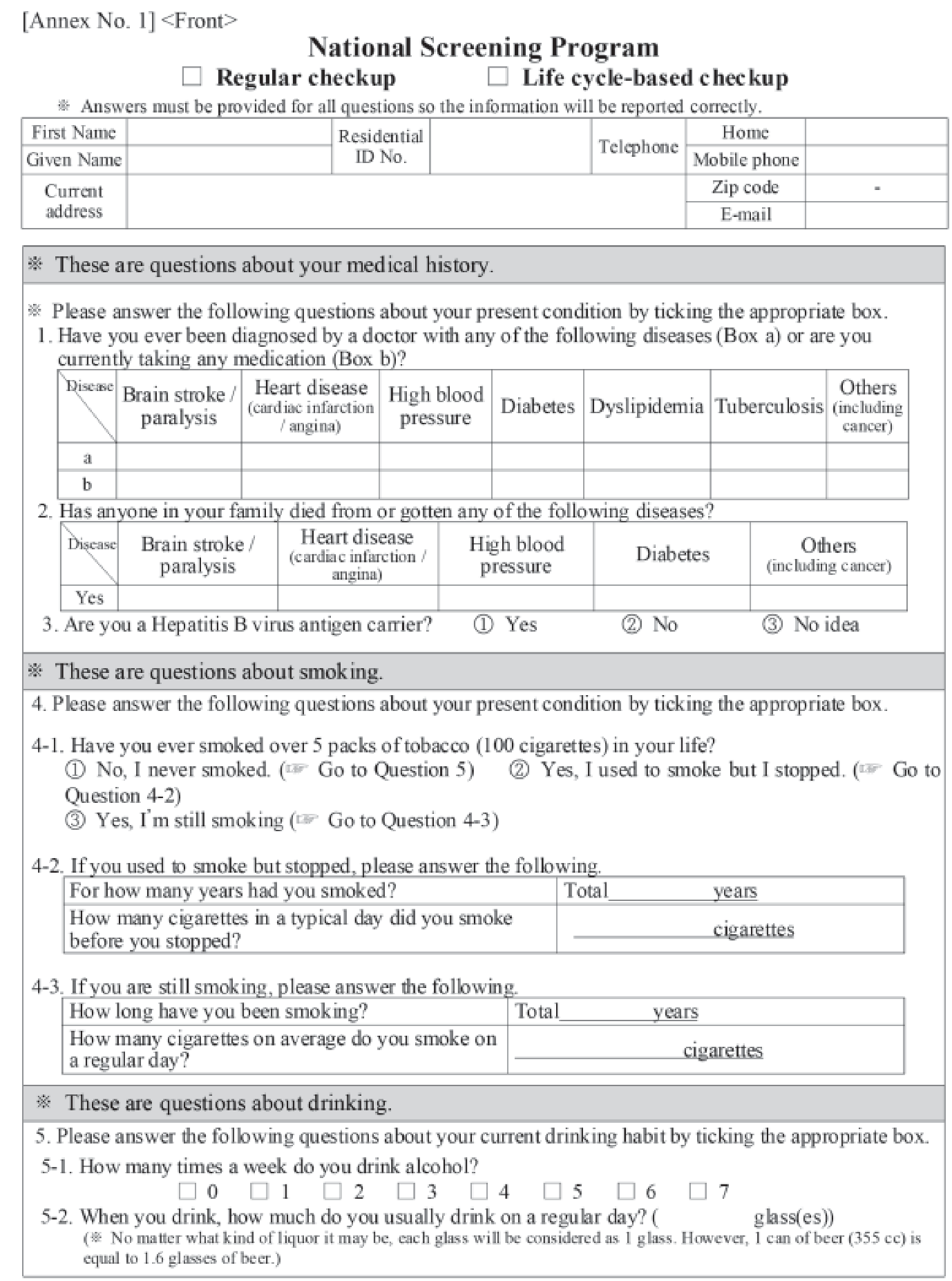


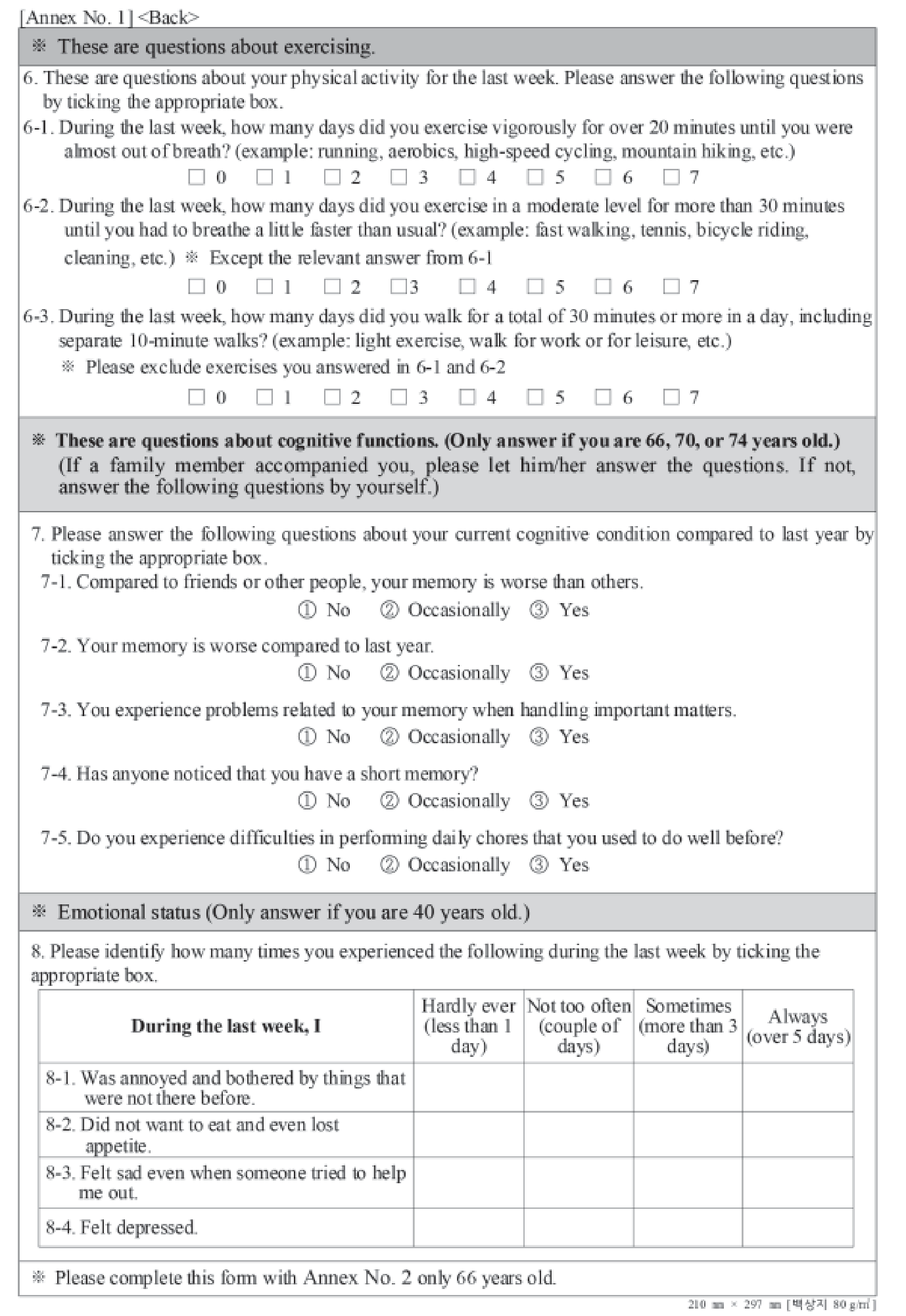


This questionnaire has been reproduced from a previously published open-access material, “Physical Activity Frequency and the Risk of Stroke: A Nationwide Cohort Study in Korea. J Am Heart Assoc. 2017;6.”, with permission from the authors.

**S2 Method. Additional information regarding the methods.**

**Details regarding data collection and variable definition**

***Characteristics collected at S1 and S3***

The following information was collected for baseline characteristics: age; sex; body mass index; waist circumference; systolic and diastolic blood pressure; fasting glucose, total cholesterol, high-density lipoprotein cholesterol, triglyceride, and serum creatinine levels; estimated glomerular filtration rate; and alanine transaminase, aspartate transaminase, and hemoglobin levels. The low-income group included those who were in the lowest quartile of the nation’s income according to the insurance fee, which is determined by the information including ones’ income. The underlying comorbidity burden was reflected by the Charlson Comorbidity Index.

The other lifestyle information included self-reported average frequencies of moderate-to-vigorous physical activity per day and alcohol consumption behavior. Moderate-to-vigorous physical activity was collected as a categorical variable: none, 1 or 2 days per week, 3 or 4 days per week, and ≥ 5 days per week. Alcohol consumption behavior was categorized as none, moderate consumption (≤ 2 standard drinks for men and ≤ 1 standard drink for women per single drinking session), and heavy consumption (more than moderate).

The underlying severity of MetS was determined according to the number of MetS components present and the actual measured values of the components in the MetS criteria. Even within a study group with the same MetS presence or absence state, the burden of MetS components may differ. The two definitions were complementary because the number of preexisting MetS components did not reflect the continuous information (e.g., high glucose values) in each MetS parameter, and the actual values of the MetS parameters could be affected by medications.

***Definition of MACEs***

MACEs were the composite outcome and included acute myocardial infarction, coronary revascularization, and acute ischemic stroke events. Acute myocardial infarction was defined as an admission event with an ICD-10 diagnostic code of I21 or I22. Coronary revascularization was defined according to the procedure history identified in the claims database. Acute ischemic stroke was defined as an admission event with an ICD-10 diagnostic code of I63.

**Details regarding statistical analysis**

***Analysis of the association between smoking status at S1 and the odds of development of or recovery from previous MetS.***

Multivariable logistic regression analysis was performed. The smoking status at S1 was the exposure variable, and the adjustment variables were also collected from S1. The first multivariable model was adjusted for age and sex. The second multivariable model was adjusted for age, sex, baseline estimated glomerular filtration rate (continuous, mL/min/1.73 m^2^), alanine aminotransferase level (continuous, IU/mL aspartate aminotransferase level (continuous, IU/mL), hemoglobin level (continuous, g/dL), low income status (the lowest quartile in the nation), body mass index, Charlson Comorbidity Index, and the severity of MetS as determined by the values of MetS components. The third multivariable model used the number of MetS components to reflect the baseline severity of MetS at S1 and the lifestyle variables [self-reported frequencies of moderate-to-vigorous physical activity (categorical, none, 1 or 2 days per week, 3 or 4 days per week, ≥ 5 days per week) and alcohol consumption behavior [none, moderate consumption (≤ 2 standard drinks for men and ≤ 1 standard drink for women per single drinking session), and heavy consumption (more than moderate)], which were additionally adjusted for.

In the component analysis, the unadjusted model and the age- and sex-adjusted model were constructed first. Next, an additional multivariable model was constructed that included age, sex, baseline estimated glomerular filtration rate (continuous, mL/min/1.73 m2), alanine aminotransferase levels (continuous, IU/mL aspartate aminotransferase levels (continuous, IU/mL), hemoglobin levels (continuous, g/dL), low income status (the lowest quartile in the nation), body mass index, and Charlson Comorbidity Index, and lifestyle variables [self-reported frequencies of moderate-to-vigorous physical activity (categorical, none, 1 or 2 days per week, 3 or 4 days per week, ≥ 5 days per week) and alcohol consumption behavior [none, moderate consumption (≤ 2 standard drinks for men and ≤ 1 standard drink for women per single drinking session), and heavy consumption (more than moderate)]. MetS severity was not adjusted for because this analysis was performed to assess associations within individual MetS components.

***Analysis of the association between smoking status at S3 and future MACE risks***

Kaplan-Meier survival curves were plotted to demonstrate the MACE outcomes in each study group according to smoking status, by the PROC LIFETEST command in SAS. The incidence rate ratios for MACE risks are shown according to smoking status at S3 in groups with different MetS statuses and were investigated after multivariable adjustment using Poisson regression. First, the age- and sex-adjusted incidence rate ratios were calculated using the nonsmokers in the MetS-free group as the reference group. The aim of this analysis was to quantitatively assess the risk of MACE according to dynamic MetS and smoking status, and other attributes were not adjusted at this time as other characteristics may represent the distinct traits of each MetS group (e.g., a higher body mass index in those with MetS). Next, additional adjustment for diverse clinicodemographic characteristics was performed to assess the associations between smoking status and MACE risk within each subgroup with nonsmokers used as the reference group. In this analysis, age, sex, baseline estimated glomerular filtration rate (continuous, mL/min/1.73 m^2^), alanine aminotransferase levels (continuous, IU/mL aspartate aminotransferase levels (continuous, IU/mL), hemoglobin levels (continuous, g/dL), low income status (the lowest quartile in the nation), body mass index, the Charlson Comorbidity Index, and the severity of MetS were adjusted. The final model was constructed with additional adjustment for the other lifestyle variables: self-reported frequencies of moderate-to-vigorous physical activity (categorical, none, 1 or 2 days per week, 3 or 4 days per week, ≥ 5 days per week) and alcohol consumption behavior [none, moderate consumption (≤ 2 standard drinks for men and ≤ 1 standard drink for women per single drinking session), and heavy consumption (more than moderate)].

**S3 Table. Characteristics of the study population at the third health screening (S3).**

| **Variables** | **Nonsmoker** | **Ex-smoker** | **Current, light-to-moderate** | **Current, heavy** |
| --- | --- | --- | --- | --- |
| **Number of people** | 3553108 | 984813 | 929468 | 632328 |
| **Clinical and demographic characteristics at S3** |  |  |  |  |
| Age (years) | 47.8±13.9 | 48.7±12.7 | 40.9±11.7 | 44.4±11.0 |
| Sex (male) | 1073809 (30) | 947245 (96) | 875179 (94) | 626363 (99) |
| Height (cm) | 161.0±8.6 | 169.8±6.5 | 170.9±6.9 | 170.7±6.3 |
| Weight (kg) | 60.2±10.6 | 69.9±10.2 | 69.3±11.3 | 70.2±11.3 |
| BMI (kg/m^2^) | 23.2±3.2 | 24.2±2.9 | 23.7±3.2 | 24.1±3.3 |
| Low-income status* | 749551 (21) | 131200 (13) | 116612 (13) | 91677 (15) |
| CCI (score) | 0.8±1.2 | 0.8±1.2 | 0.5±0.9 | 0.6±1.1 |
| Hemoglobin (g/dL) | 13.5±1.5 | 14.9±1.2 | 15.1±1.2 | 15.3±1.2 |
| AST (IU/L) | 21.6±20.9 | 28.2±24.5 | 28.0±28.6 | 29.6±25.4 |
| ALT (IU/L) | 23.7±16.13 | 26.9±19.3 | 26.0±20.0 | 27.3±21.7 |
| Cr (mg/dL) | 0.82±0.18 | 0.97±0.16 | 0.97±0.16 | 0.97±0.16 |
| eGFR (mL/min/1.73 m^2^) | 92.1±30.8 | 90.1±36.1 | 94.0±38.5 | 93.5±37.4 |
| **Self-reported lifestyle at S3** |  |  |  |  |
| Moderate-to-vigorous activity |  |  |  |  |
| None | 1781453 (50) | 356257 (36) | 380992 (41) | 302456 (48) |
| 1-2 days/wk | 971124 (27) | 342513 (35) | 348225 (37) | 207360 (33) |
| 3-4 days/wk | 486333 (14) | 180293 (18) | 131090 (14) | 77657 (12) |
| ≥ 5 days/wk | 314198 (9) | 105750 (11) | 69161 (7) | 44855 (7) |
| Alcohol |  |  |  |  |
| No alcohol intake | 2243470 (63) | 284865 (29) | 215518 (23) | 154404 (24) |
| Moderate consumption | 175203 (5) | 61686 (6) | 44552 (5) | 25778 (4) |
| Heavy consumption | 1134435 (32) | 638262 (65) | 669398 (72) | 452146 (72) |
| **Parameters of MetS at S3** |  |  |  |  |
| Waist circumference (cm) | 77.3±9.0 | 83.4±7.7 | 81.5±8.2 | 83.2±8.2 |
| Systolic BP (mmHg) | 119.2±14.3 | 123.6±13.4 | 121.6±13.0 | 123.1±13.4 |
| Diastolic BP (mmHg) | 74.3±9.5 | 77.4±9.3 | 76.5±9.2 | 77.5±9.4 |
| Glucose (mg/dL) | 94.9±19.2 | 99.0±22.1 | 95.5±20.7 | 99.4±26.4 |
| Triglycerides (mg/dL) | 110.3±78.7 | 140.5±108.2 | 147.6±117.1 | 168.8±136.7 |
| HDL cholesterol (mg/dL) | 58.2±14.8 | 53.9±14.6 | 53.8±14.8 | 52.0±15.2 |
| **N of MetS components in S3** |  |  |  |  |
| 0 | 1219701 (34) | 234892 (24) | 281935 (30) | 142886 (23) |
| 1 | 1015172 (29) | 299251 (30) | 300965 (32) | 189364 (30) |
| 2 | 627783 (18) | 224449 (23) | 202459 (22) | 150636 (24) |
| 3 | 282363 (8) | 104412 (11) | 74206 (8) | 72155 (11) |
| 4 | 266479 (8) | 87375 (9) | 52510 (6) | 56820 (9) |
| 5 | 141610 (4) | 34434 (4) | 17393 (2) | 20467 (3) |

MetS = metabolic syndrome, BMI = body mass index, CCI = Charlson Comorbidity Index, AST = aspartate aminotransferase, ALT = alanine aminotransferase, Cr = creatinine, eGFR = estimated glomerular filtration rate, BP = blood pressure, HDL = high-density lipoprotein

There were no missing values in the table.

**S4 Table. Association of smoking status and the development of a component of MetS in individuals who were free from the component at the first health screening (S1).**

| Prior MetS status and outcome | Smoking status at S1 | Subjects without the component at S1 | Number of cases with development of the component (percent) | Unadjusted model | | Model 1. Age-/sex-adjusted | | ^a^Model 2. Clinical factors including other adjusted lifestyle variables | |
| --- | --- | --- | --- | --- | --- | --- | --- | --- | --- |
|  |  |  |  | OR (95% CI) | P | Adjusted OR (95% CI) | P | Adjusted OR (95% CI) | P |
| High triglycerides | Nonsmoker | 2608750 | 352689 (14) | Reference |  | Reference |  | Reference |  |
|  | Ex-smoker | 505615 | 98407 (19) | 1.55 (1.53-1.56) | <0.001 | 1.10 (1.09-1.11) | <0.001 | 1.07 (1.06-1.08) | <0.001 |
|  | Current, light-to-moderate | 579355 | 122444 (21) | 1.71 (1.70-1.73) | <0.001 | 1.53 (1.52-1.54) | <0.001 | 1.58 (1.56-1.59) | <0.001 |
|  | Current, heavy | 362724 | 95302 (26) | 2.28 (2.26-2.3) | <0.001 | 1.78 (1.76-1.79) | <0.001 | 1.79 (1.78-1.81) | <0.001 |
| Low HDL | Nonsmoker | 2543137 | 385214 (15) | Reference |  | Reference |  | Reference |  |
|  | Ex-smoker | 678693 | 79262 (12) | 0.74 (0.73-0.75) | <0.001 | 1.05 (1.04-1.06) | <0.001 | 1.08 (1.07-1.09) | <0.001 |
|  | Current, light-to-moderate | 779649 | 73077 (9) | 0.58 (0.57-0.58) | <0.001 | 1.11 (1.10-1.12) | <0.001 | 1.20 (1.19-1.21) | <0.001 |
|  | Current, heavy | 550205 | 71885 (13) | 0.84 (0.83-0.85) | <0.001 | 1.44 (1.42-1.45) | <0.001 | 1.50 (1.48-1.51) | <0.001 |
| Impaired glucose tolerance | Nonsmoker | 2485778 | 408126 (16) | Reference |  | Reference |  | Reference |  |
|  | Ex-smoker | 506435 | 117486 (23) | 1.54 (1.53-1.55) | <0.001 | 1.11 (1.10-1.12) | <0.001 | 1.05 (1.04-1.06) | <0.001 |
|  | Current, light-to-moderate | 643623 | 124103 (19) | 1.22 (1.21-1.22) | <0.001 | 1.13 (1.12-1.14) | <0.001 | 1.10 (1.09-1.11) | <0.001 |
|  | Current, heavy | 429164 | 106714 (25) | 1.69 (1.67-1.70) | <0.001 | 1.35 (1.33-1.36) | <0.001 | 1.27 (1.26-1.29) | <0.001 |
| Elevated BP | Nonsmoker | 2068347 | 412675 (20) | Reference |  | Reference |  | Reference |  |
|  | Ex-smoker | 386701 | 117370 (30) | 1.75 (1.74-1.76) | <0.001 | 1.02 (1.01-1.03) | <0.001 | 0.95 (0.94-0.96) | <0.001 |
|  | Current, light-to-moderate | 515346 | 140904 (27) | 1.51 (1.50-1.52) | <0.001 | 1.13 (1.12-1.14) | <0.001 | 1.09 (1.08-1.10) | <0.001 |
|  | Current, heavy | 344041 | 109049 (32) | 1.86 (1.85-1.88) | <0.001 | 1.15 (1.14-1.16) | <0.001 | 1.06 (1.05-1.07) | <0.001 |
| Central obesity | Nonsmoker | 2596974 | 288588 (11) | Reference |  | Reference |  | Reference |  |
|  | Ex-smoker | 663763 | 56866 (9) | 0.75 (0.74-0.76) | <0.001 | 1.05 (1.04-1.07) | <0.001 | 1.00 (0.99-1.01) | 0.99 |
|  | Current, light-to-moderate | 787574 | 59460 (8) | 0.65 (0.65-0.66) | <0.001 | 1.10 (1.09-1.12) | <0.001 | 1.20 (1.19-1.22) | <0.001 |
|  | Current, heavy | 550605 | 50050 (9) | 0.80 (0.79-0.81) | <0.001 | 1.26 (1.25-1.28) | <0.001 | 1.30 (1.29-1.32) | <0.001 |

MetS = metabolic syndrome, OR = odds ratio, CI = confidence interval

Heavy smoking was defined as smoking ≥ 20 cigarettes per day.

^a^Model 2 was adjusted for age, sex, baseline eGFR (continuous, mL/min/1.73 m2), alanine aminotransferase (continuous, IU/mL aspartate aminotransferase (continuous, IU/mL), hemoglobin (continuous, g/dL), low income status (the lowest quartile in the nation), body mass index (continuous, kg/m2), the Charlson Comorbidity Index, and the values of MetS components and other lifestyle variables [self-reported frequencies of moderate-to-vigorous exercise (categorical: none/week, 1-2 days/week, 3-4 days/week, and ≥ 5 days/week) and drinking status (categorical: none; moderate consumption, ≤ 2 standard drink for male and ≤ 1 for female subjects per single drinking session; and heavy consumption as more than moderate)] at the first health screening (S1).

**S5 Table. Association between smoking status and recovery from a component of MetS in individuals previously with the component at the first health screening (S1).**

| Prior MetS status and outcome | Smoking status at S1 | Subjects with the component at S1 | Number of cases with recovery from the component (percent) | Unadjusted model | | Model 1. Age-/sex-adjusted | | ^a^Model 2. Clinical factors including other lifestyle variables adjusted | |
| --- | --- | --- | --- | --- | --- | --- | --- | --- | --- |
|  |  |  |  | OR (95% CI) | P | Adjusted OR (95% CI) | P | Adjusted OR (95% CI) | P |
| High triglycerides | Nonsmoker | 736375 | 283070 (38) | Reference |  | Reference |  | Reference |  |
|  | Ex-smoker | 280532 | 101489 (36) | 0.91 (0.90-0.92) | <0.001 | 0.86 (0.85-0.87) | <0.001 | 0.92 (0.91-0.93) | <0.001 |
|  | Current, light-to-moderate | 277575 | 104759 (38) | 0.97 (0.96-0.98) | <0.001 | 0.73 (0.72-0.74) | <0.001 | 0.72 (0.71-0.73) | <0.001 |
|  | Current, heavy | 275814 | 86071 (31) | 0.73 (0.72-0.73) | <0.001 | 0.60 (0.59-0.60) | <0.001 | 0.62 (0.61-0.62) | <0.001 |
| Low HDL | Nonsmoker | 776427 | 344921 (44) | Reference |  | Reference |  | Reference |  |
|  | Ex-smoker | 138255 | 63387 (46) | 1.06 (1.05-1.07) | <0.001 | 0.88 (0.87-0.89) | <0.001 | 0.90 (0.88-0.91) | <0.001 |
|  | Current, light-to-moderate | 120073 | 66618 (55) | 1.56 (1.54-1.58) | <0.001 | 0.93 (0.92-0.95) | <0.001 | 0.93 (0.91-0.94) | <0.001 |
|  | Current, heavy | 117045 | 57400 (49) | 1.20 (1.19-1.22) | <0.001 | 0.79 (0.78-0.81) | <0.001 | 0.83 (0.81-0.84) | <0.001 |
| Impaired glucose tolerance | Nonsmoker | 763483 | 375296 (49) | Reference |  | Reference |  | Reference |  |
|  | Ex-smoker | 260833 | 111461 (43) | 0.77 (0.76-0.78) | <0.001 | 0.87 (0.86-0.88) | <0.001 | 0.95 (0.94-0.96) | <0.001 |
|  | Current, light-to-moderate | 206465 | 113791 (55) | 1.27 (1.26-1.28) | <0.001 | 0.97 (0.96-0.99) | <0.001 | 0.99 (0.98-1.00) | 0.11 |
|  | Current, heavy | 201380 | 90675 (45) | 0.85 (0.84-0.86) | <0.001 | 0.75 (0.74-0.76) | <0.001 | 0.82 (0.81-0.83) | <0.001 |
| Elevated BP | Nonsmoker | 1177614 | 371441 (32) | Reference |  | Reference |  | Reference |  |
|  | Ex-smoker | 378762 | 111521 (29) | 0.91 (0.9-0.91) | <0.001 | 0.90 (0.90-0.91) | <0.001 | 1.00 (0.99-1.01) | 0.49 |
|  | Current, light-to-moderate | 315753 | 133961 (42) | 1.60 (1.59-1.61) | <0.001 | 0.96 (0.96-0.97) | <0.001 | 1.00 (0.99-1.01) | 0.99 |
|  | Current, heavy | 279709 | 97881 (35) | 1.17 (1.16-1.18) | <0.001 | 0.87 (0.86-0.88) | <0.001 | 0.96 (0.95-0.97) | <0.001 |
| Central obesity | Nonsmoker | 760534 | 257662 (34) | Reference |  | Reference |  | Reference |  |
|  | Ex-smoker | 154629 | 58894 (38) | 1.20 (1.19-1.21) | <0.001 | 1.03 (1.02-1.04) | <0.001 | 1.01 (0.99-1.03) | 0.22 |
|  | Current, light-to-moderate | 124433 | 43684 (35) | 1.06 (1.04-1.07) | <0.001 | 0.78 (0.77-0.80) | <0.001 | 0.8 (0.78-0.81) | <0.001 |
|  | Current, heavy | 127805 | 40704 (32) | 0.91 (0.90-0.92) | <0.001 | 0.70 (0.69-0.71) | <0.001 | 0.74 (0.72-0.75) | <0.001 |

MetS = metabolic syndrome, OR = odds ratio, CI = confidence interval

Heavy smoking was defined as smoking ≥ 20 cigarettes per day.

^a^Model 2 was adjusted for age, sex, baseline eGFR (continuous, mL/min/1.73 m2), alanine aminotransferase levels (continuous, IU/mL aspartate aminotransferase (continuous, IU/mL), hemoglobin levels (continuous, g/dL), low income status (the lowest quartile in the nation), body mass index (continuous, kg/m2), the Charlson Comorbidity Index, and the values of MetS components and other lifestyle variables [self-reported frequencies of moderate-to-vigorous exercise (categorical: none/week, 1-2 days/week, 3-4 days/week, and ≥ 5 days/week) and drinking status (categorical: none; moderate consumption, ≤ 2 standard drink for male and ≤ 1 for female subjects per single drinking session; and heavy consumption as more than moderate)] at the first health screening (S1).

**S6 Table. Risk of acute myocardial infarction according to smoking status at the third health screening (S3) and dynamic metabolic syndrome status.**

| MetS status | Smoking status at S3 | Number of persons | Number of acute MI | Follow-up (person-years) | Incidence rate (/1000 person-years) | Unadjusted model | | Age-/sex-adjusted | |
| --- | --- | --- | --- | --- | --- | --- | --- | --- | --- |
|  |  |  |  |  |  | IRR (95% CI) | P | Adjusted IRR (95% CI) | P |
| MetS-free | Nonsmoker | 2683410 | 5233 | 11587438 | 0.45 | 1 (Ref.) | < 0.001 | 1 (Ref.) | < 0.001 |
|  | Ex-smoker | 691272 | 2212 | 3119304 | 0.71 | 1.57 (1.49-1.65) | < 0.001 | 1.15 (1.09-1.21) | < 0.001 |
|  | Current, light-to-moderate | 730020 | 2052 | 3324731 | 0.62 | 1.37 (1.30-1.44) | < 0.001 | 1.62 (1.53-1.71) | < 0.001 |
|  | Current, heavy | 437113 | 2029 | 1994115 | 1.02 | 2.25 (2.14-2.37) | < 0.001 | 2.21 (2.09-2.34) | < 0.001 |
| MetS-recovery | Nonsmoker | 179246 | 866 | 749040 | 1.16 | 2.56 (2.38-2.75) | < 0.001 | 1.49 (1.39-1.60) | < 0.001 |
|  | Ex-smoker | 67320 | 360 | 298000 | 1.21 | 2.67 (2.40-2.98) | < 0.001 | 1.54 (1.38-1.72) | < 0.001 |
|  | Current, light-to-moderate | 55339 | 315 | 249434 | 1.26 | 2.80 (2.50-3.13) | < 0.001 | 2.47 (2.20-2.77) | < 0.001 |
|  | Current, heavy | 45773 | 397 | 205848 | 1.93 | 4.27 (3.86-4.73) | < 0.001 | 3.54 (3.19-3.92) | < 0.001 |
| MetS-developed | Nonsmoker | 190217 | 1160 | 761886 | 1.52 | 3.37 (3.16-3.59) | < 0.001 | 1.66 (1.56-1.77) | < 0.001 |
|  | Ex-smoker | 65811 | 455 | 281987 | 1.61 | 3.57 (3.25-3.93) | < 0.001 | 1.83 (1.66-2.02) | < 0.001 |
|  | Current, light-to-moderate | 47207 | 353 | 206241 | 1.71 | 3.79 (3.40-4.22) | < 0.001 | 3.08 (2.76-3.44) | < 0.001 |
|  | Current, heavy | 44392 | 444 | 193887 | 2.29 | 5.07 (4.60-5.59) | < 0.001 | 4.00 (3.62-4.42) | < 0.001 |
| MetS-chronic | Nonsmoker | 500235 | 4125 | 1977354 | 2.09 | 4.62 (4.43-4.81) | < 0.001 | 1.91 (1.83-2.00) | < 0.001 |
|  | Ex-smoker | 160410 | 1323 | 683130 | 1.94 | 4.29 (4.04-4.55) | < 0.001 | 1.94 (1.82-2.07) | < 0.001 |
|  | Current, light-to-moderate | 96902 | 1017 | 421703 | 2.41 | 5.34 (4.99-5.71) | < 0.001 | 3.64 (3.39-3.90) | < 0.001 |
|  | Current, heavy | 105050 | 1339 | 458713 | 2.92 | 6.46 (6.09-6.86) | < 0.001 | 4.51 (4.23-4.81) | < 0.001 |

MetS = metabolic syndrome, MI = myocardial infarction, IRR = incidence rate ratio, CI = confidence interval

Heavy smoking was defined as smoking ≥ 20 cigarettes per day.

**S7 Table. Risk of coronary revascularization according to smoking at the third health screening (S3) and dynamic metabolic syndrome status.**

| MetS status | Smoking status at S3 | Number of persons | Number of revasculari--zations | Follow-up (person-years) | Incidence rate (/1000 person-years) | Unadjusted model | | Age-/sex-adjusted | |
| --- | --- | --- | --- | --- | --- | --- | --- | --- | --- |
|  |  |  |  |  |  | IRR (95% CI) | P | Adjusted IRR (95% CI) | P |
| MetS-free | Nonsmoker | 2683410 | 3490 | 11587438 | 0.30 | 1 (Ref.) | < 0.001 | 1 (Ref.) | < 0.001 |
|  | Ex-smoker | 691272 | 2556 | 3119304 | 0.82 | 2.72 (2.59-2.86) | < 0.001 | 1.36 (1.29-1.44) | < 0.001 |
|  | Current, light-to-moderate | 730020 | 1753 | 3324731 | 0.53 | 1.75 (1.65-1.85) | < 0.001 | 1.53 (1.44-1.62) | < 0.001 |
|  | Current, heavy | 437113 | 1957 | 1994115 | 0.98 | 3.26 (3.08-3.44) | < 0.001 | 2.29 (2.16-2.43) | < 0.001 |
| MetS-recovery | Nonsmoker | 179246 | 760 | 749040 | 1.01 | 3.37 (3.11-3.64) | < 0.001 | 1.88 (1.74-2.04) | < 0.001 |
|  | Ex-smoker | 67320 | 526 | 298000 | 1.77 | 5.86 (5.35-6.42) | < 0.001 | 2.27 (2.07-2.49) | < 0.001 |
|  | Current, light-to-moderate | 55339 | 335 | 249434 | 1.34 | 4.46 (3.99-4.99) | < 0.001 | 2.80 (2.50-3.14) | < 0.001 |
|  | Current, heavy | 45773 | 426 | 205848 | 2.07 | 6.87 (6.21-7.60) | < 0.001 | 4.02 (3.63-4.45) | < 0.001 |
| MetS-developed | Nonsmoker | 190217 | 1181 | 761886 | 1.55 | 5.15 (4.82-5.50) | < 0.001 | 2.56 (2.39-2.73) | < 0.001 |
|  | Ex-smoker | 65811 | 716 | 281987 | 2.54 | 8.43 (7.78-9.14) | < 0.001 | 2.87 (2.65-3.12) | < 0.001 |
|  | Current, light-to-moderate | 47207 | 399 | 206241 | 1.93 | 6.42 (5.79-7.12) | < 0.001 | 3.73 (3.36-4.14) | < 0.001 |
|  | Current, heavy | 44392 | 539 | 193887 | 2.78 | 9.23 (8.43-10.11) | < 0.001 | 5.13 (4.68-5.62) | < 0.001 |
| MetS-chronic | Nonsmoker | 500235 | 4833 | 1977354 | 2.44 | 8.12 (7.77-8.48) | < 0.001 | 3.48 (3.32-3.64) | < 0.001 |
|  | Ex-smoker | 160410 | 2466 | 683130 | 3.61 | 11.99 (11.38-12.62) | < 0.001 | 3.59 (3.40-3.79) | < 0.001 |
|  | Current, light-to-moderate | 96902 | 1401 | 421703 | 3.32 | 11.03 (10.37-11.74) | < 0.001 | 5.32 (4.99-5.67) | < 0.001 |
|  | Current, heavy | 105050 | 1925 | 458713 | 4.20 | 13.93 (13.18-14.73) | < 0.001 | 6.79 (6.41-7.19) | < 0.001 |

MetS = metabolic syndrome, MACE = major adverse cardiovascular events, IRR = incidence rate ratio, CI = confidence interval

Heavy smoking was defined as smoking ≥ 20 cigarettes per day.

**S8 Table. Risk of acute ischemic stroke according to smoking at the third health screening (S3) and dynamic metabolic syndrome status.**

| MetS status | Smoking status at S3 | Number of persons | Number of acute ischemic stroke | Follow-up (person-years) | Incidence rate (/1000 person-years) | Unadjusted model | | Age-/sex-adjusted | |
| --- | --- | --- | --- | --- | --- | --- | --- | --- | --- |
|  |  |  |  |  |  | IRR (95% CI) | P | Adjusted IRR (95% CI) | P |
| MetS-free | Nonsmoker | 2683410 | 9435 | 11587438 | 0.81 | 1 (Ref.) | < 0.001 | 1 (Ref.) | < 0.001 |
|  | Ex-smoker | 691272 | 3540 | 3119304 | 1.13 | 1.39 (1.34-1.45) | < 0.001 | 0.99 (0.95-1.03) | 0.661 |
|  | Current, light-to-moderate | 730020 | 2872 | 3324731 | 0.86 | 1.06 (1.02-1.11) | 0.006 | 1.46 (1.39-1.52) | < 0.001 |
|  | Current, heavy | 437113 | 2598 | 1994115 | 1.30 | 1.60 (1.53-1.67) | < 0.001 | 1.80 (1.71-1.88) | < 0.001 |
| MetS-recovery | Nonsmoker | 179246 | 1683 | 749040 | 2.25 | 2.76 (2.62-2.91) | < 0.001 | 1.37 (1.3-1.44) | < 0.001 |
|  | Ex-smoker | 67320 | 656 | 298000 | 2.20 | 2.70 (2.50-2.93) | < 0.001 | 1.43 (1.32-1.55) | < 0.001 |
|  | Current, light-to-moderate | 55339 | 437 | 249434 | 1.75 | 2.15 (1.95-2.37) | < 0.001 | 1.99 (1.81-2.19) | < 0.001 |
|  | Current, heavy | 45773 | 480 | 205848 | 2.33 | 2.86 (2.61-3.14) | < 0.001 | 2.58 (2.36-2.84) | < 0.001 |
| MetS-developed | Nonsmoker | 190217 | 2296 | 761886 | 3.01 | 3.70 (3.54-3.87) | < 0.001 | 1.49 (1.42-1.56) | < 0.001 |
|  | Ex-smoker | 65811 | 769 | 281987 | 2.73 | 3.35 (3.11-3.60) | < 0.001 | 1.52 (1.41-1.64) | < 0.001 |
|  | Current, light-to-moderate | 47207 | 468 | 206241 | 2.27 | 2.79 (2.54-3.06) | < 0.001 | 2.31 (2.1-2.53) | < 0.001 |
|  | Current, heavy | 44392 | 574 | 193887 | 2.96 | 3.64 (3.34-3.96) | < 0.001 | 3.07 (2.82-3.35) | < 0.001 |
| MetS-chronic | Nonsmoker | 500235 | 8749 | 1977354 | 4.42 | 5.43 (5.28-5.59) | < 0.001 | 1.74 (1.69-1.79) | < 0.001 |
|  | Ex-smoker | 160410 | 2344 | 683130 | 3.43 | 4.21 (4.03-4.41) | < 0.001 | 1.65 (1.57-1.73) | < 0.001 |
|  | Current, light-to-moderate | 96902 | 1367 | 421703 | 3.24 | 3.98 (3.76-4.21) | < 0.001 | 2.63 (2.48-2.79) | < 0.001 |
|  | Current, heavy | 105050 | 1788 | 458713 | 3.90 | 4.79 (4.55-5.04) | < 0.001 | 3.46 (3.28-3.64) | < 0.001 |

MetS = metabolic syndrome, MACE = major adverse cardiovascular events, IRR = incidence rate ratio, CI = confidence interval

Heavy smoking was defined as smoking ≥ 20 cigarettes per day.
